# Myeloproliferative Neoplasm-like Mutations of Calreticulin Induce Phenotypes Associated with Calreticulin Dysfunction in *C. elegans*

**DOI:** 10.3390/ijms252111606

**Published:** 2024-10-29

**Authors:** Ana Guijarro-Hernández, Cristina Hurtado, Estibaliz Urizar-Compains, Begoña Ezcurra, Alberto Galiana-Sáenz, Enrique Baquero, Juan Cabello, José Luis Vizmanos

**Affiliations:** 1 Department of Biochemistry and Genetics, School of Sciences, University of Navarra, 31008 Pamplona, Spain; aguijarro@alumni.unav.es (A.G.-H.); churtado@unav.es (C.H.); eurizarc@unav.es (E.U.-C.); agalianasae@alumni.unav.es (A.G.-S.); 2 Center for Biomedical Research of La Rioja (CIBIR), 26006 Logroño, Spain; bezcurra@riojasalud.es (B.E.); juan.cabello@riojasalud.es (J.C.); 3 Department of Environmental Biology, School of Sciences, University of Navarra, 31008 Pamplona, Spain; ebaquero@unav.es; 4 Institute for Biodiversity and Environment BIOMA, University of Navarra, 31008 Pamplona, Spain

**Keywords:** calreticulin, myeloproliferative neoplasms, loss of function, development, molting, *Caenorhabditis elegans*

## Abstract

In previous research, we created a *C. elegans* model with homozygous mutations in calreticulin similar to those found in patients with essential thrombocythemia (ET) and primary myelofibrosis (PMF), two myeloproliferative neoplasms (MPNs). This model, lacking JAK orthologs, enabled us to examine the transcriptomic effects caused by mutant calreticulin without the influence of JAK/STAT activation, the primary pathogenic mechanism associated with calreticulin mutations known to date. Most of the gene expression changes observed seemed to be due to a partial loss of protein function, with the alteration of the extracellular matrix being particularly notable. In this study, our aim was to determine whether this model exhibited any phenotype related to these transcriptomic alterations. The results demonstrate that these strains exhibit multiple phenotypes related to the alteration of the extracellular matrix, fat levels, and fertility, which could be a possible consequence of a partial loss of calreticulin function. These phenotypes resemble some of the clinical and molecular characteristics described in patients with MPNs, but they had never before been linked to a loss of protein function in humans. Thus, these results collectively suggest that *CALR* mutations could have significant effects on MPNs due to loss of protein function. Delving deeper into these effects to develop innovative therapies for these patients offers considerable potential and interest, given that targeted therapies for these patients have not yielded very promising results so far.

## 1. Introduction

The nematode *Caenorhabditis elegans* has made significant contributions to our understanding of the molecular mechanisms involved in cancer development, including processes like apoptosis [1]. Likewise, this organism has helped uncover the link between hypoxia and apoptosis in tumor progression [2] and has provided important insights into the roles of autophagy, the cell cycle, and other processes involved in cancer development [3,4,5].

The approach to study cancer in *C. elegans* is very particular since this organism is incapable of generating malignant tumors [1]. However, this limitation can be overcome by comparative analyses of phenotypes. Orthologous phenotypes, or phenologs, are defined as the phenotypes arising from the disruption of equivalent gene orthologs in two organisms. For example, mutations in the genes of the *him* family produce a high incidence of males in *C. elegans*, while alterations in their human orthologs (*TP53* and *BRCA1*) are implicated in several types of cancer. Thus, the identification of orthologous phenotypes results in a list of genes associated with the phenotype, from which potential molecular mechanisms involved in the disease can be identified [1,6].

In this study, we used the model organism *C. elegans* to investigate myeloproliferative neoplasms (MPNs), a group of rare blood cancers characterized by the clonal expansion of mature myeloid cells. Our work was focused on the study of essential thrombocythemia (ET) and primary myelofibrosis (PMF), two MPNs that can be caused by mutations in *CALR*, the gene encoding calreticulin.

Patients with ET may present with a broad spectrum of symptoms, ranging from asymptomatic cases to complications associated with thrombosis, bleeding, and vasomotor disturbances. PMF patients typically experience symptoms such as night sweat, fatigue, weight loss, and pruritus. Moreover, PMF is characterized by extramedullary hematopoiesis and fibrosis of the bone marrow. The gene harboring the driver mutation plays a significant role in shaping the clinical presentation. Thus, *CALR* mutations have been associated with higher platelet counts in ET patients and an increased risk of transformation to fibrosis, which is one of the main causes of mortality among individuals with ET [7].

The mechanism by which *CALR* mutations induce myeloproliferation remains unclear. It has been reported that mutant CALR binds to the extracellular domain of MPL in the endoplasmic reticulum, resulting in dimerization, transport to the cell surface, and activation of the JAK2/STAT pathway [7,8,9]. Additionally, a recent study has suggested that the activation of the JAK2/STAT signaling pathway in *CALR*-mutated cells may also occur in an MPL-independent manner [10]. In any case, although a role for the JAK2/STAT pathway in MPNs has been emphasized repeatedly, there are certain complexities in this relationship. The presence of an inflammatory state in MPNs may complicate the understanding of the role of the JAK2/STAT pathway in these disorders, as inflammation and cytokine production can influence the activation of the pathway in ways that are not specific to MPNs [7].

In this context, there is a growing emphasis on investigating alternative mechanisms independent of JAK2/STAT activation that may elucidate the reasons why mutations in *CALR* result in myeloproliferation. To date, several mechanisms triggered by mutant CALR that are independent of JAK2/STAT activation have been identified (reviewed in [11]). First, mutant CALR appears to cause resistance to UPR-induced apoptosis and genomic instability by downregulating OXR1 in K562 cells [12]. A recent study also demonstrated that *CALR* mutations impair calreticulin function, leading to weakened responses to oxidative stress and DNA damage [10], a result previously suggested by our *C. elegans* model with calreticulin mutations [13]. Additionally, *CALR* mutations increase protein secretion and bind to CALR receptors on antigen-presenting cells, limiting their ability to phagocytize cancer cells that express wild-type CALR and promoting immune evasion [14]. This effect is further exacerbated by decreased binding affinity for PDIA3, resulting in a loss of function in the peptide loading complex, which is responsible for loading cellular antigens onto MHC-I molecules [15,16]. It has also been reported that defective interaction between mutant CALR and the SOCE machinery triggers TPOR-independent cytosolic calcium fluxes in megakaryocytes that lead to their uncontrolled proliferation [17]. Finally, various studies suggest that *CALR* mutations promote tumorigenesis by modulating transcription through interactions with nuclear transcription factors [15,18], and other reports support their role in activating key pathways of MPNs, including Hedgehog signaling, in a JAK2/STAT-independent manner across different models [10,13].

Despite the advances made in the molecular description of the pathogenesis of *CALR*-mutated ET and PMF, so far it has not been possible to develop any targeted therapy capable of curing these diseases or reducing the mutant allele burden in these patients. Currently, the only approved targeted therapy for patients with myelofibrotic-phase ET and for non-transplanted patients with PMF is the JAK1/JAK2 inhibitor ruxolitinib, which aims to decrease the activation of the JAK2/STAT pathway [19]. However, this therapy does not appear to be more effective than other second-line treatments for ET [20] and only palliates symptoms such as splenomegaly in patients with PMF [19]. Therefore, studying JAK2/STAT-independent mechanisms derived from mutant calreticulin could be of great interest, as it could lead to the discovery of new therapeutic targets in order to develop new treatment approaches or to improve the efficacy of existing therapies based on JAK inhibitors.

In this context, *C. elegans* seems to be a suitable organism for studying the JAK2/STAT-independent pathogenic mechanisms of mutant calreticulin since it shows an ortholog of calreticulin (*crt-1*) and lacks orthologs of JAK proteins. In this way, the introduction of mutations similar to those causing myeloproliferative neoplasms in humans could allow the identification of the JAK2/STAT-independent effects of mutant calreticulin.

Expanding upon this logic, we created two strains of *C. elegans* with mutations in homozygosity in *crt-1* (*crt-1*(*knu378*) and *crt-1*(*jvp1*)) in a previous study [13]. These mutations, respectively, recreate those known as type 1 and type 2 mutations, typically found in heterozygosity in the calreticulin of patients with MPNs [13]. Transcriptomic analyses of these nematodes allowed us to elucidate some possible altered mechanisms resulting from mutant calreticulin [13]. Most of the observed expression changes appeared to correspond to a partial loss of protein function, with the alteration of the extracellular matrix being particularly notable. In the present work, we aim to study whether *crt-1*(*knu378*) and *crt-1*(*jvp1*) mutants exhibit any phenotype related to the transcriptomic alteration of the extracellular matrix (development, molting, and morphology and resistance of the cuticle), as well as other phenotypes previously associated with the loss of function of *crt-1* in *C. elegans* (fertility, response to endoplasmic reticulum (ER) stress, and regulation of lipid homeostasis) [21,22] that could be related to some of the alterations that emerged in the transcriptomic analyses. The description of these phenotypes can help to identify new mechanisms that may be key in the development or progression of *CALR*-mutated ET and PMF and point to new possible therapeutic targets for these diseases.

## 2. Results

### 2.1. crt-1 Mutations Delay Larval Development and Affect Molting

**Figure 1:**
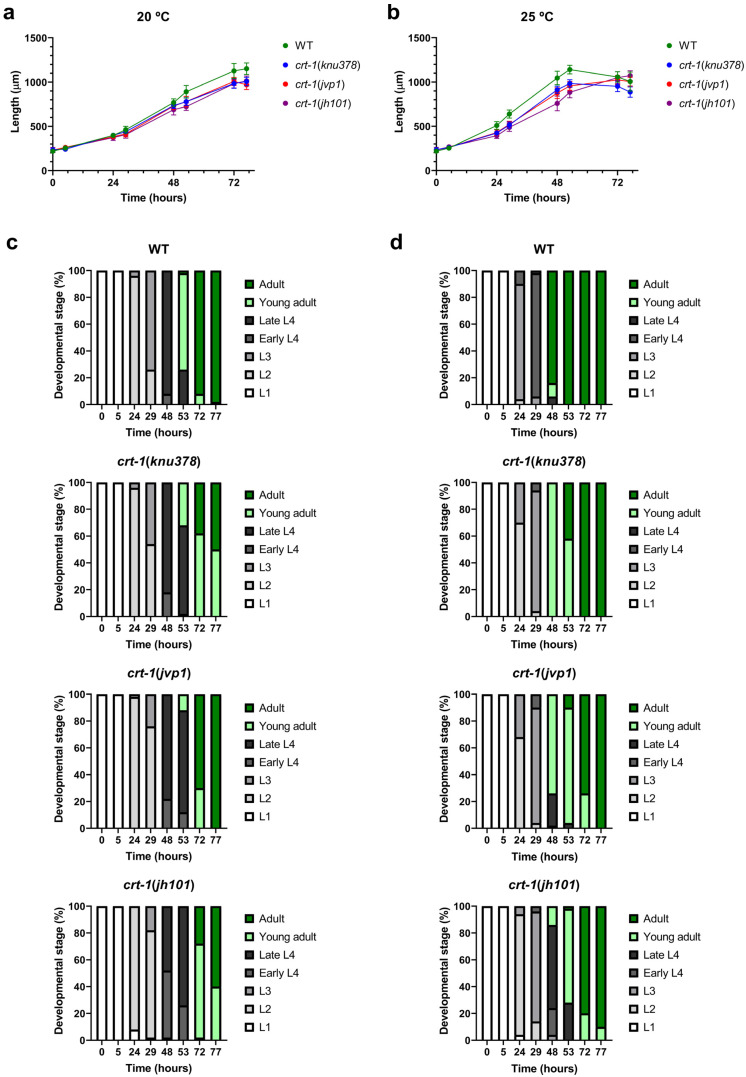
Larval development at 20 and 25 °C. *crt-1* mutations delay larval development at 20 and 25 °C. (**a**,**b**) Length of individuals during growth at 20 and 25 °C, respectively. The mean lengths obtained for 50 worms per strain at 0, 5, 24, 29, 48, 53, 72, and 77 h are represented, along with their standard deviation. (**c**,**d**) Classification into larval stages of the individuals at 20 and 25 °C, respectively. The percentage of worms in each larval stage (L1, L2, L3, early L4, late L4, young adult, or adult) out of a total of 50 worms assessed per strain at 0, 5, 24, 29, 48, 53, 72, and 77 h is represented. The results of the statistical analysis conducted for these assays are detailed in Figures S1–S4.

The mutant strains in *crt-1* exhibited a temporal growth delay in length compared to the wild-type strain under both baseline conditions (20 °C) (Figure 1a) and conditions that slightly induce overexpression of *crt-1* (25 °C) [21] (Figure 1b). The growth delay was very similar between *crt-1*(*knu378*) and *crt-1*(*jvp1*) worms and lower than that observed for the *crt-1*(*jh101*) strain at both temperatures (Figure 1a,b). Differences between *crt-1*(*knu378*)/*crt-1*(*jvp1*) worms and the *crt-1*(*jh101*) strain were more pronounced between 29 and 53 h at 20 °C (Figure S1) and between 24 and 53 h at 25 °C (Figure S2).

To determine if the growth delay in terms of length was also associated with a developmental delay, the larval stage of the worms was evaluated at each of the time points studied based on their morphological characteristics. The results showed that the *crt-1* mutant strains also took longer than the wild-type strain to reach various larval stages, both under baseline conditions (20 °C) (Figure 1c) and under conditions that slightly induce the overexpression of *crt-1* (25 °C) (Figure 1d). This delay in development was similar to that observed with length and was also very similar between *crt-1*(*knu378*) and *crt-1*(*jvp1*) worms and less than that observed for the *crt-1*(*jh101*) strain at both temperatures (Figure 1c,d). Also, as in the case of length, the differences between *crt-1*(*knu378*)/*crt-1*(*jvp1*) worms and the *crt-1*(*jh101*) strain were more noticeable between 29 and 53 h at 20 °C (Figure S3) and between 24 and 53 h at 25 °C (Figure S4).

As expected, all strains grew faster at 25 °C than at 20 °C, both in length and larval development. However, at 25 °C, the differences between strains were greater (Figure 1b,d).

**Figure 2:**
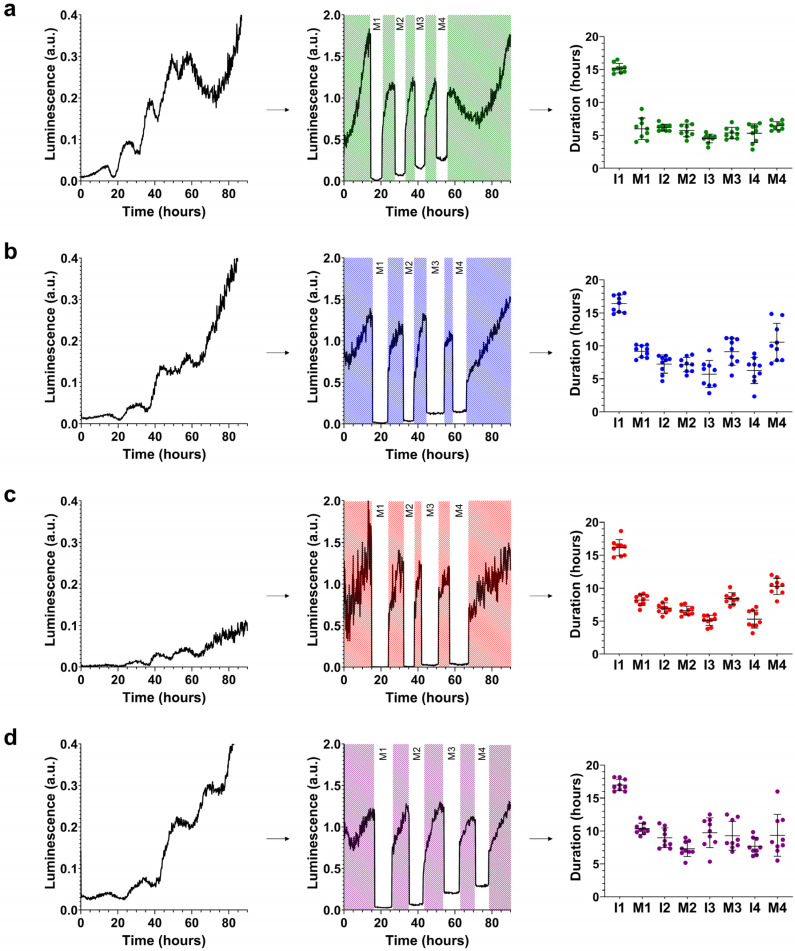
Molting patterns at 20 °C. *crt-1* mutants show aberrant molting patterns at 20 °C. The molting pattern of each strain was studied using bioluminescence. On the left side of each panel, the raw bioluminescence values obtained are represented. In the middle part, the corrected interphase values are represented by dividing the raw data of the interphases by the mean of each interphase while maintaining the raw bioluminescence values obtained in each molt (M; in white). On the right side, the duration values of each interphase (I) and molt (M) are represented, along with their mean and standard deviation (*n* = 9). Results corresponding to (**a**) wild-type strain, (**b**) *crt-1*(*knu378*), (**c**) *crt-1*(*jvp1*), and (**d**) *crt-1*(*jh101*). The results of the statistical analysis conducted for this assay are detailed in Figure 3.

The analysis of molting at 20 °C confirmed that the *crt-1*(*knu378*) and *crt-1*(*jvp1*) strains exhibited a very similar larval development, and in both cases, it was intermediate to that observed in the wild-type and *crt-1*(*jh101*) strains (Figure 2).

**Figure 3:**
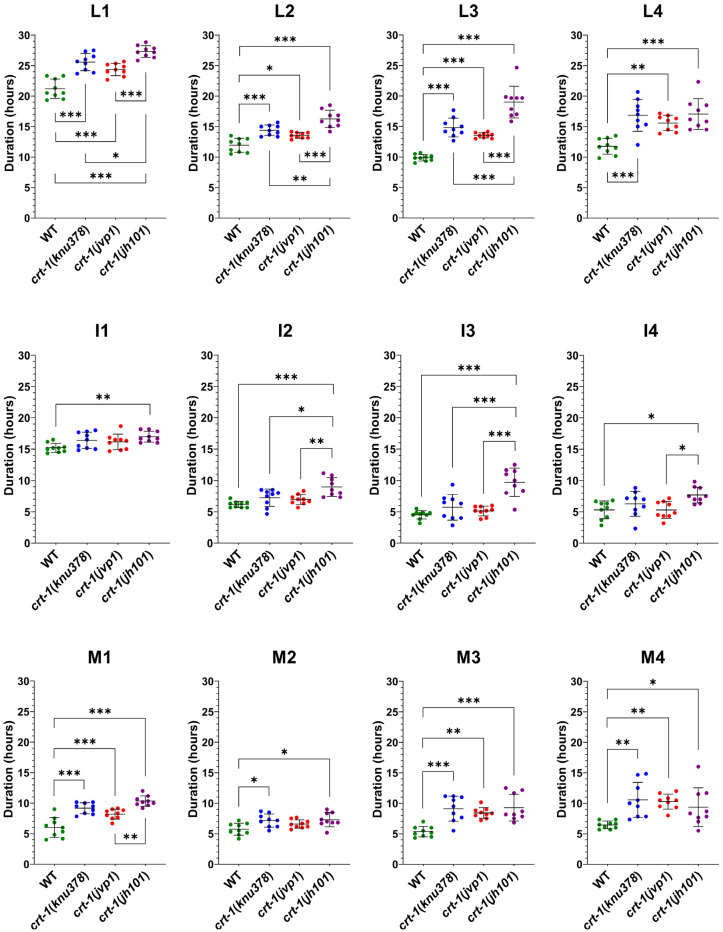
Molting duration at 20 °C. *crt-1* mutants exhibit longer molts than those of the wild-type strain, and the interphases of *crt-1*(*jh101*) worms are larger than those of *crt-1*(*knu378*) and *crt-1*(*jvp1*) mutants. The duration of the larval stage (L), interphase (I), and molt (M) at 20 °C is represented. The duration of the larval stage is the sum of the duration of the interphase and the molt. Individual data (*n* = 9), mean, and standard deviation are represented. Differences were considered significant (*) when *p* < 0.05, very significant (**) when *p* < 0.01, and highly significant (***) when *p* < 0.001.

Specifically, the duration of L1, L2, and L3 stages in *crt-1*(*knu378*) and *crt-1*(*jvp1*) worms was longer than that observed for the wild-type strain and shorter than that of the *crt-1*(*jh101*) strain (Figure 3). The length of the L4 stage in *crt-1*(*knu378)*, *crt-1*(*jvp1*), and *crt-1*(*jh101*) worms was also longer than that of the wild-type strain, but in this case, no differences were found between the *crt-1* mutant strains (Figure 3). Worms from the mutant strains in *crt-1* exhibited molts (M1, M2, M3, and M4) that were very similar to each other and longer than those of the wild-type strain, causing the delay in the development of the L1, L2, L3, and L4 stages compared to the wild-type strain (Figure 3). In general, the *crt-1*(*jh101*) strain showed longer interphases than the wild-type strain and *crt-1*(*knu378*) and *crt-1*(*jvp1*) worms. In the three latter strains, the length of interphases I1, I2, I3, and I4 was very similar (Figure 3).

### 2.2. crt-1 Mutants Show Cuticle Defects Without Major Alterations of the Hypodermal Cell Lineage

**Figure 4:**
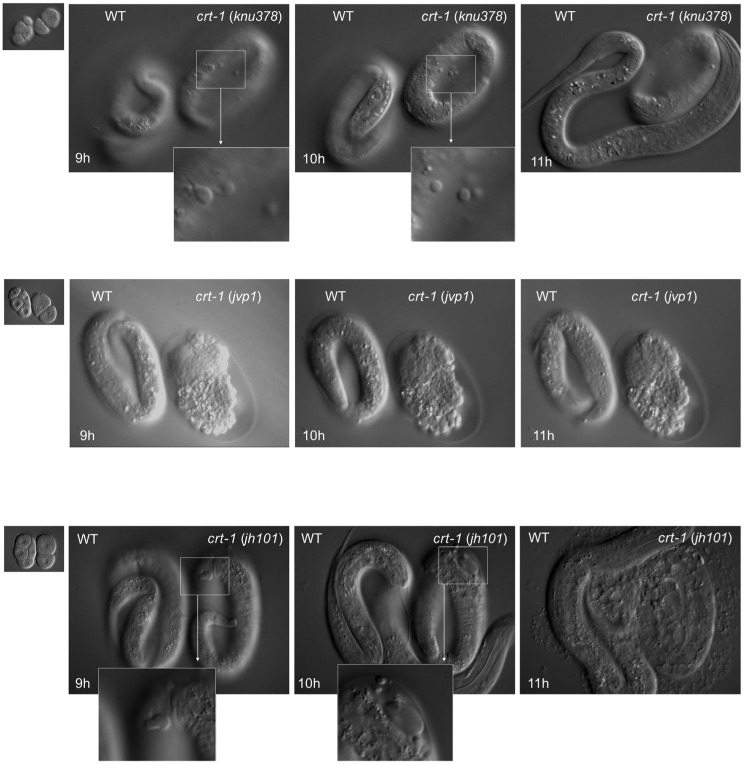
Embryo development. *crt-1* mutants show cuticle defects during embryo development. Differential interference contrast still images (time 0, 9 h, 10 h, and 11 h) of developing wild-type and *crt-1* mutant embryos analyzed using 4D microscopy. *crt-1*(*knu378*) are shown in the **top row**, *crt-1*(*jvp1*) in the **middle row**, and *crt-1*(*jh101*) in the **bottom row**. Details of cuticle blebbing are highlighted for *crt-1*(*knu378*) and *crt-1*(*jh101*). Example of *crt-1*(*jvp1*) (**middle panel**) that died due to ventral enclosure defect, while other specimens also show cuticle blebbing during development.

To assess the possible function of CRT-1 during embryonic development, we performed 4D microscopy analysis of *crt-1*(*knu378*), *crt-1*(*jvp1*), and *crt-1*(*jh101*) mutant embryo development and compared them to wild-type controls. To ensure the embryos developed under the exact same conditions and that there were no experimental variations from wild-types to mutants, we recorded a mutant and a wild-type embryo side-by-side in the same preparation for each case. For each genotype, the recordings were performed in triplicate to score the penetrance of the phenotype. In all cases, we found a reproducible effect of the *ctr-1* mutation on hypodermic cells and the embryonic cuticle. Two embryos, one *crt-1*(*jvp1*) and one *crt-1*(*jh101*), died during development due to an incomplete ventral closure of the hypodermal cells (ventral closure defect) and the subsequent extrusion of internal tissues (Figure 4). As for the rest of the embryos analyzed, three *crt-1*(*knu378*), two *crt-1*(*jvp1*), and two *crt-1*(*jh101*) developed normally but showed notable cuticle blebbing after 7–9 h of development as early as the three-fold stage. The bleb size was larger in *crt-1*(*jh101*) embryos than in *crt-1*(*knu378*) or *crt-1*(*jvp1*) embryos, and one of the *crt-1*(*jh101*) exploded after 10 h of development (Figure 4).

**Figure 5:**
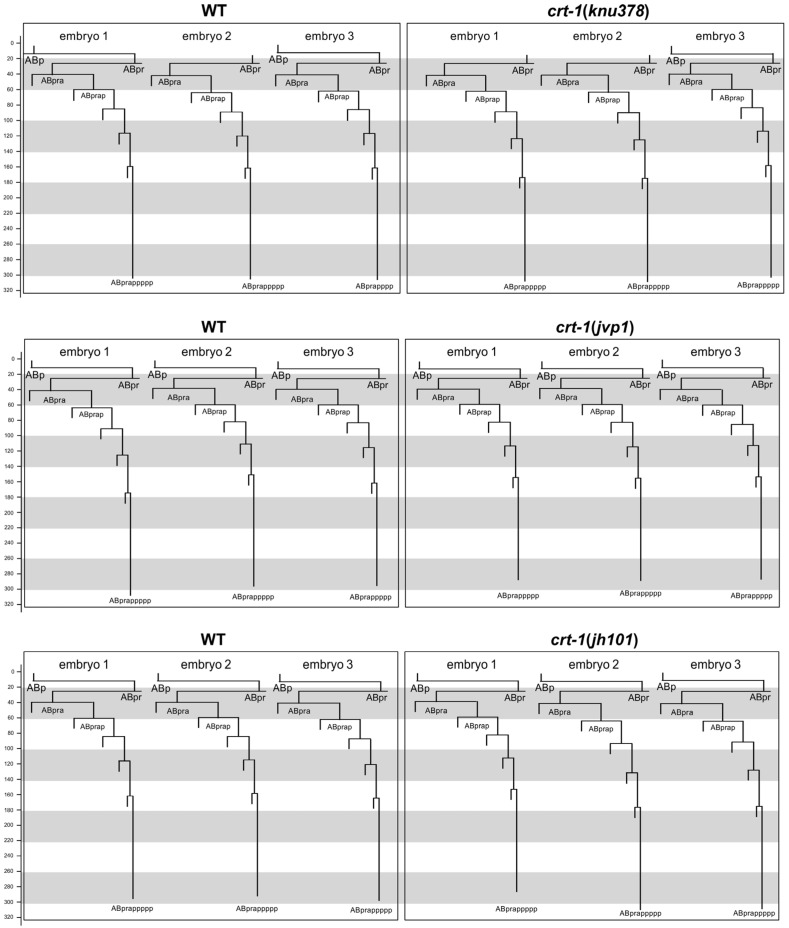
Embryo development. Cell lineage of a hypodermal cell (ABpra) in wild-type and *crt-1* mutant embryos. **Top panels** show the comparison between three wild-type embryos (**left**) and three *crt-1*(*knu378*) mutant embryos (**right**). **Middle panels** show the comparison between three wild-type embryos (**left**) and three *crt-1*(*jvp1*) mutant embryos (**right**). **Bottom panels** show the comparison between three wild-type embryos (**left**) and three *crt-1*(*jh101*) mutant embryos (**right**). Timing is shown in minutes. The cleavage pattern of wild-type hypodermis is generally conserved in *ctr-1* mutants. Cell lineage was traced up to 320 min; cells cannot be followed beyond this time due to embryo movement.

To determine whether the cuticle blebbing and ventral closure defects of *crt-1* mutants were a consequence of an error in the fate specification of the hypodermis during development, we took advantage of the invariant *C. elegans* embryo cell lineage. We reconstructed the cell lineage of ABpra blastomere descendants fated to differentiate as hypodermal cells. Cell lineage analysis of *crt-1* mutant vs. wild-type embryos did not identify any abnormal cell division pattern beyond a slight delay in the timing of late cell division (Figure 5). This delay in late development is further supported by the fact that none of the mutants hatched during the 12 h that the recordings lasted, while the corresponding paired wild-types did.

Altogether, these results highlight a function of CRT-1 in embryonic hypodermal cells (responsible for cuticle secretion) independent of fate specification. In addition, given its phenotype and that it is a true loss-of-function deletion allele, *crt-1*(*jh101*) might be considered the strongest allele.

**Figure 6:**
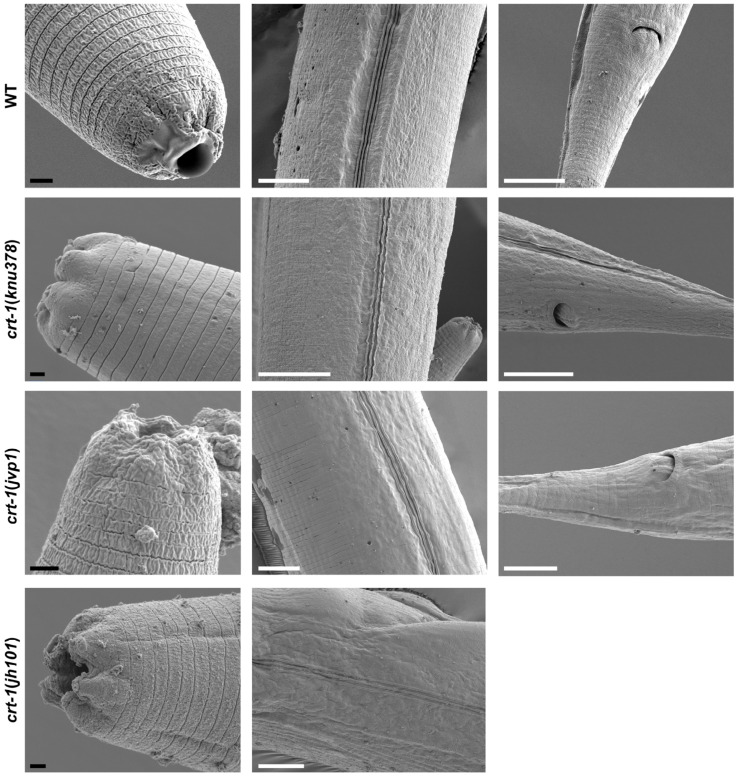
Images of the cuticle obtained by FE-SEM from the mouth, body, and tail regions. *crt-1* mutants do not show any morphological alteration of the cuticle in the adult stage. The images of the mouth, body, and tail are taken at magnifications of 10,000–20,000×, 4000×, and 4000–6000×, respectively. The black scale bar corresponds to 1 μm and the white scale bar to 10 μm.

**Figure 7:**
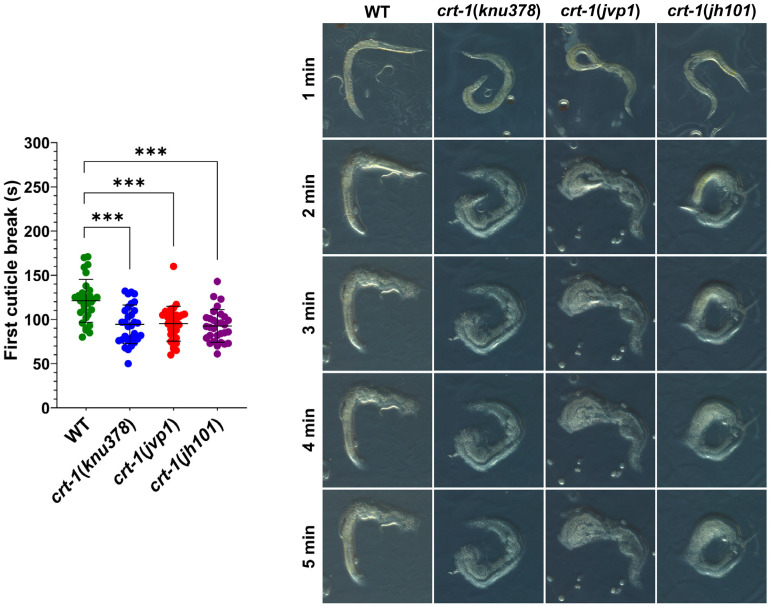
Cuticle resistance. *crt-1* mutants show lower resistance of the cuticle than the wild-type strain. Individual values of the first cuticle breakage after exposure to sodium hypochlorite with 6–14% active chlorine for 30 worms per strain are represented, along with the mean and standard deviation. Differences were considered highly significant (***) when *p* < 0.001. Representative images of the behavior of each of the evaluated strains taken at 13.5× magnifications after 1, 2, 3, 4, and 5 min of exposure to the chemical product are included.

Observation by FE-SEM microscopy showed that the cuticle of adult *crt-1* mutants did not show any notable defects compared to the wild-type strain in the mouth, body, or tail regions (Figure 6). The alae and annuli, key structures of the cuticle, were particularly examined, but no differences were detected between the strains. However, adults from *crt-1* mutant strains exhibited a less resistant cuticle compared to wild-type strains when exposed to sodium hypochlorite (Figure 7).

### 2.3. crt-1(knu378) and crt-1(jvp1) Mutants Exhibit Some Phenotypes Consistent with the Loss of Function of CRT-1

**Figure 8:**
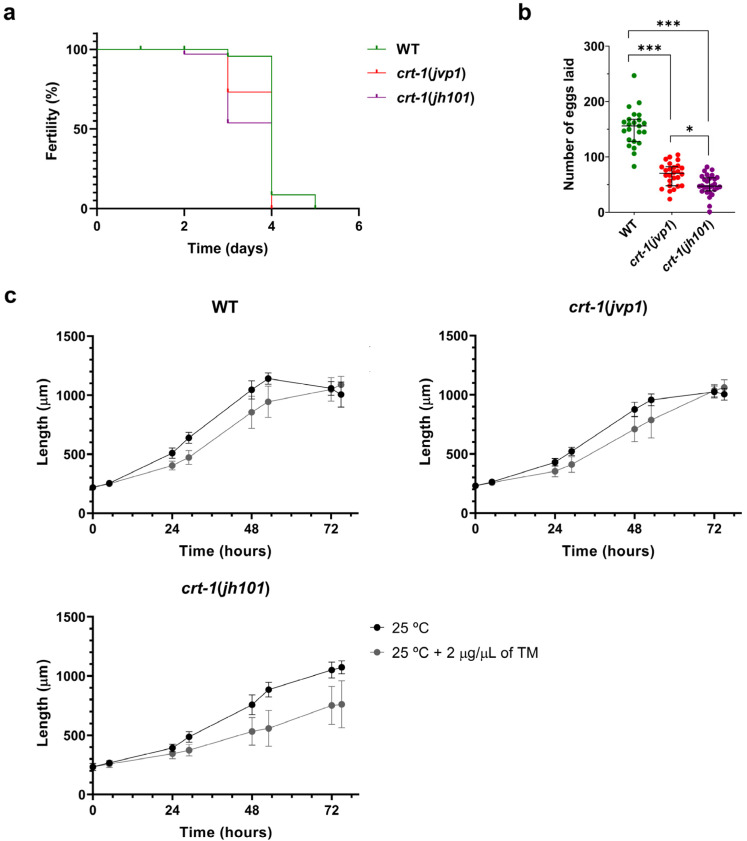
Fertility and resistance to ER stress. *crt-1*(*jvp1*) mutants have reduced fertility similar to strain *crt-1*(*jh101*), but they withstand ER stress better than *crt-1*(*jh101*) worms. (**a**) Kaplan–Meier curve representing the day on which the strains cease to lay eggs at 25 °C (*n* = 36). (**b**) Number of eggs laid by each of the strains throughout their fertile lifespan (*n* = 36). Differences were considered significant (*) when *p* < 0.05, and highly significant (***) when *p* < 0.001. (**c**) Growth in length of *crt-1* mutants at 25 °C and 25 °C when 2 μg/μL of tunicamycin was added to the medium. In all cases, the mean lengths obtained for 50 worms per strain at 0, 5, 24, 29, 48, 53, 72, and 77 h are represented, along with their standard deviation.

Individuals from the *crt-1*(*jvp1*) and *crt-1*(*jh101*) strains exhibited a decrease in fertility compared to individuals from the wild-type strain at 25 °C. Firstly, worms from the *crt-1* mutant strains lost the ability to self-fertilize and lay eggs prior to worms from the wild-type strain (Figure 8a). Moreover, worms from the *crt-1* mutant strains laid fewer eggs throughout their fertile life compared to the wild-type strain. In this case, slight differences were observed between the *crt-1*(*jvp1*) strain and the *crt-1*(*jh101*) strain, with the number of eggs laid by individuals of the latter strain being lower (Figure 8b).

Although individuals from the wild-type, *crt-1*(*jvp1*), and *crt-1*(*jh101*) strains were impaired in development at 25 °C after exposure to ER stress induced by 2 μg/μL tunicamycin (Figure 8c), the effects were not uniform between individuals of different strains. Regarding individual length data, a significant dispersion of the data was observed in the three strains between 48 and 53 h of growth. However, from 72 h onward, dispersion persisted only in the *crt-1*(*jh101*) strain (Figure S5). Regarding larval development, it was observed that most of the worms of the *crt-1*(*jh101*) strain were arrested between the L1 and L4 stages. In contrast, worms of the wild-type and *crt-1*(*jvp1*) strains mostly reached the adult stage (Figure S6). Therefore, it seems that the *crt-1*(*jh101*) strain is the most affected by the presence of 2 μg/μL of tunicamycin, while the *crt-1*(*jvp1*) strain was affected similarly to the wild-type, indicating it does not behave like a loss-of-function allele in this case.

**Figure 9:**
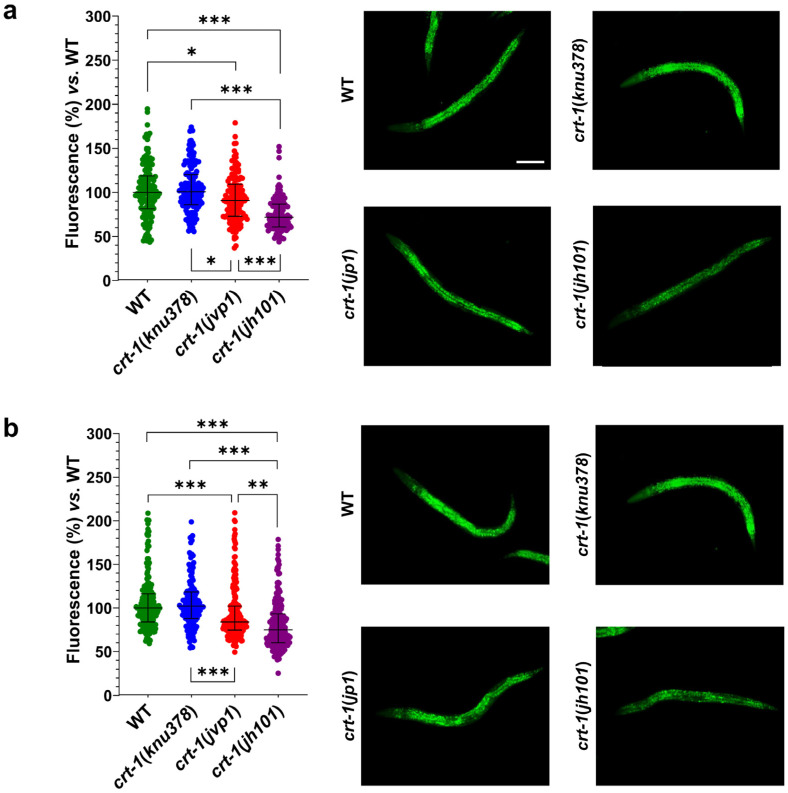
Fat levels with Nile Red staining. *crt-1*(*jvp1*) and *crt-1*(*jh101*) mutants exhibit lower fat levels than the wild-type strain at 20 °C and 25 °C. (**a**,**b**) Results of Nile Red staining of worms developed at 20 and 25 °C, respectively. In both cases, the (**left**) percentage of fluorescence emitted after staining of 127–214 L4 worms per strain and temperature with Nile Red is represented compared to the mean fluorescence emitted by stained wild-type worms. The statistics used are the mean and standard deviation. Differences were considered significant (*) when *p* < 0.05, very significant (**) when *p* < 0.01, and highly significant (***) when *p* < 0.001. The images on the right are representative of the fluorescence emitted by each of the strains at a 10× magnification. The scale bar corresponds to 100 μm.

Regarding fat levels, *crt-1*(*knu378*) worms showed no differences compared to wild-type worms at either 20 or 25 °C. However, *crt-1*(*jvp1*) worms showed slightly lower fat levels than those of the wild-type strain at both temperatures (Figure 9). The decrease in fat compared to the wild-type strain was most pronounced at 25 °C, a temperature at which calreticulin is slightly overexpressed. Finally, *crt-1*(*jh101*) worms showed lower fat levels than those of the wild-type and *crt-1*(*jvp1*) worms at both 20 and 25 °C (Figure 9). In this case, the decrease in fat compared to the wild-type strain remained unchanged at 25 °C.

## 3. Discussion

Mutations in *CALR* are capable of inducing ET and PMF, two MPNs characterized by the clonal expansion of mature myeloid cells. Although it is well known that calreticulin mutations activate the JAK2/STAT cascade, other mechanisms not involving JAK/STAT activation that may also contribute to these diseases have been proposed for the mutant protein. These JAK/STAT-independent mechanisms have not been extensively investigated yet and could be valuable for developing new targeted therapies, given the apparent lack of efficacy of JAK inhibitors in patients.

In this context, we developed a model in *C. elegans* with mutations in calreticulin that are orthologous to those present in patients with ET and PMF (*crt-1*(*knu378*) and *crt-1*(*jvp1*)) and lacking JAK orthologs, as established in a previous study. According to published data, transcriptomic analysis of these strains revealed some possible JAK/STAT-independent mechanisms derived from mutant calreticulin [13]. Most of the observed expression changes appeared to correspond to a partial loss of protein function, with the alteration of the extracellular matrix being particularly remarkable. In this study, our aim was to investigate whether these mutant strains in *crt-1* exhibit any phenotype related to extracellular matrix alteration (development, molting, and morphology and resistance of the cuticle), as well as to assess other phenotypes previously associated with calreticulin dysfunction (fertility, response to ER stress, and regulation of lipid homeostasis). The study of these orthologous phenotypes may be useful for identifying key mechanisms that could be relevant in the pathogenesis of the disease in humans.

Firstly, we observed that strains *crt-1*(*knu378*) and *crt-1*(*jvp1*) exhibited a developmental delay. Since the hours at which differences were found between these strains are the same when comparing both the length of growth and the larval stage throughout their development, it seems that the growth delay in the length of the strains is due to a delay in their larval development. The molting assay showed that the differences in development between *crt-1* mutant strains and the wild-type strain were due to the longer molts of M1-M4. The development of the *crt-1*(*jh101*) strain, used as a control for the loss of function of calreticulin, was slower than in the strains *crt-1*(*knu378*) and *crt-1*(*jvp1*) due to longer intervals between molts.

Next, we decided to investigate whether these strains also exhibited alterations in their embryonic development. An embryo from strain *crt-1*(*knu378*) and one from strain *crt-1*(*jvp1*) died during their development due to a ventral closure defect, resulting in the extrusion of their internal tissues. Ventral closure relies on actin cytoskeleton reorganization [23], but no other defects typically associated with *ced-10*/RAC1 (involved in actin polymerization) were detected. On the other hand, most embryos analyzed from strains *crt-1*(*knu378*) and *crt-1*(*jvp1*), and all analyzed from strain *crt-1*(*jh101*), developed normally but exhibited cuticle blebbing during development, with the latter strain showing a greater extent. Subsequent experiments showed that this phenotype was not due to an error in the fate specification of the hypodermis during development. Thus, the blebbing could be caused by the cuticle being detached from the hypodermis or because its structure or composition is altered. Electron microscopy results discarded any structural alterations in the cuticle of the strains, suggesting that this defect was caused by an altered composition of the cuticle, a result supported by the altered expression of multiple cuticle components in the previously published transcriptomic study [13] or because the cuticle was detached from the hypodermis. Both hypotheses are consistent with the finding that the cuticle of *crt-1* mutant strains exhibits lower resistance to external stress than the wild-type strain.

In all the phenotypes observed, strains *crt-1*(*knu378*) and *crt-1*(*jvp1*) behaved in an intermediate manner between the wild-type strain and the strain *crt-1*(*jh101*). In *C. elegans*, development, molting, cuticle morphology, and cuticle resistance are intricately connected to the extracellular matrix. Specifically, the extracellular matrix plays a fundamental role during development by providing structural support and signaling cues that guide the differentiation and organization of tissues. Additionally, studies on molting, morphology, and resistance aim to investigate various aspects of the cuticle, which serves as a primary form of extracellular matrix in this nematode. Thus, these findings imply that all three *crt-1* mutant strains exhibit an alteration in their extracellular matrix, suggesting that this is due to a loss of function of the CRT-1 protein. This result is particularly noteworthy because patients with PMF present an alteration of the extracellular matrix in the bone marrow, which leads to fibrosis [24], and also because alterations in the centrosome have been repeatedly associated with MPNs [25]. Therefore, the disruption of the extracellular matrix in patients with PMF may be caused by the mechanisms that lead to the aforementioned phenotypic alterations in the *C. elegans* model.

Furthermore, our results support the idea that in addition to the well-known oncogenic function of mutant calreticulin in patients with ET and PMF by activating JAK/STAT signaling through its binding to the thrombopoietin receptor, there would also be a loss of function that may lead to major alterations [26]. This also suggests that the changes observed in the extracellular matrix of these patients could be a consequence of this loss of function. In fact, an important role has been described for the non-mutated version of calreticulin in the extracellular matrix and in the secretion and processing of collagen [27].

Regarding the study of other phenotypes related to the loss of function of calreticulin, the results confirmed that the strain *crt-1*(*jh101*) does not exhibit defects in the ER stress, but it does have them in fertility and lipid metabolism.

Thus, the study of ER stress at 25 °C demonstrated that the strain *crt-1*(*jh101*) is more affected by the presence of 2 μg/μL tunicamycin than the wild-type strain. On the contrary, worms from the strain *crt-1*(*jvp1*) were affected similarly to individuals from the wild-type strain. This indicates that, while calreticulin plays a role in the response to ER stress in the worm, the introduced mutations (in this case, type 2) do not seem to affect this function. These results also appear to be consistent with a recent publication indicating that while some calreticulin mutations seem to activate the IRE1α/XBP1 pathway of the Unfolded Protein Response to drive MPNs, the type 2 mutations that have been recreated in the mutant strain *crt-1*(*jvp1*) do not seem to have this effect [28].

In the fertility assay, the strain *crt-1*(*jvp1*) exhibited fertility defects similar to those of *crt-1*(*jh101*) animals. Specifically, these strains lost the ability to self-fertilize and lay eggs earlier than the wild-type strain, in addition to decreasing the number of eggs laid during their fertile lifespan. In the latter case, minor differences were observed between both strains, which again would be compatible with a partial loss of function of the mutant calreticulin. Previously, it has been reported that calreticulin may be important for the proper development of sperm and oocytes in *C. elegans* [21], so the *crt-1*(*jvp1*) animals could have a defect in the development of both, leading to reduced fertility. Regarding this assay, we find it inappropriate to establish an orthologous phenotype in humans because the mutations in patients are somatic and, therefore, do not affect the germline or gonads. Thus, this result simply verifies that the introduction of mutations similar to those found in MPN patients leads to a loss-of-function effect; therefore, the mutations in humans could also have corresponding effects related to a loss of function of the protein.

Finally, the study of fat levels revealed that strain *crt-1*(*knu378*) behaves similarly to the wild-type strain, while *crt-1*(*jvp1*) and *crt-1*(*jh101*) animals exhibit lower fat levels than wild types. Fat levels in *crt-1*(*jh101*) mutants were lower than those in *crt-1*(*jvp1*) worms, again suggesting a partial loss of function of calreticulin in *crt-1*(*jvp1*). In support of our findings, it has been recently described that the loss of calreticulin regulates lipid homeostasis by altering the Ca^2+^ levels in the ER in mice. However, this article describes that the loss of calreticulin results in an increase in neutral lipid levels in both mice and *C. elegans* [22]. The differences in the results obtained in the present study could be due to intrinsic differences between the capacities of dyes to stain fat stores in live and fixed worms [29]. It is well known that lipid metabolism is important in hematopoiesis and, more specifically, in myeloproliferative neoplasms. In fact, it has been described that hematopoietic cells with defective cholesterol efflux in a hypercholesterolemic environment are noted to phenocopy MPNs [30]. Therefore, it does not seem unreasonable to think that lipid alterations in *CALR*-mutated patients with MPNs may be due, at least in part, to a loss of function of the mutant calreticulin.

As mentioned earlier, some of the phenotypes observed in this study have also been found in patients, but for many of them, it had not been previously proposed that the mechanism by which they are triggered could be a loss of function of calreticulin. This may have occurred because the majority of studies have been conducted in mice, which could have posed a limitation for the analysis of loss-of-function effects since calreticulin deficiency is lethal [31], unlike in *C. elegans*. Although it has been described that these mice overcome embryonic lethality and survive to term through the expression of calcineurin in the heart [32], this model remains suboptimal for studying JAK/STAT-independent mechanisms of mutant calreticulin due to the presence of JAK orthologs, contrary to what occurs in *C. elegans*. The *C. elegans* model employed in this study not only allows mutations to be homozygous without being lethal but also enables the clear observation of loss-of-function effects, which are undoubtedly independent of JAK/STAT activation, as there are no JAK orthologs. Thus, the use of *C. elegans* in this study has allowed for verification that many of the phenotypes triggered by type 1 and type 2 mutations of calreticulin could be due to a loss of function because they behave similarly to the calreticulin-deficient strain.

This finding presents an opportunity to explore calreticulin loss of function as a potential therapeutic target for MPN patients, either as a standalone approach or in combination with existing JAK inhibitors. A logical initial strategy could involve delivering a functional copy of the *CALR* gene to the patient’s cells. Although gene therapy has shown promise in treating other diseases, its application in MPNs has not yet been developed. Additionally, providing a non-mutated version of calreticulin directly is currently not a feasible option due to the challenges associated with protein delivery, intracellular localization, and ensuring proper functionality. In the near term, it may be more practical to explore therapies that target extracellular matrix components or antifibrotic agents, as well as treatments aimed at mitigating ER stress and oxidative stress resulting from calreticulin loss of function.

## 4. Materials and Methods

### 4.1. C. elegans Strains and Maintenance

To evaluate the effects of patient-like calreticulin mutations, the strains COP1358 (*crt-1*(*knu378*)) and JLV544 (*crt-1*(*jvp1*)) were used. Both were obtained using CRISPR/Cas9 technology in a previous study [13] and recreate type 1 and type 2 mutations, respectively. Additionally, the wild-type strain *Bristol* N2 and the *crt-1* null mutant strain KJ216 (*crt-1*(*jh101*)) were used as controls of the normal function of calreticulin and the complete loss of function of the protein, respectively. Both strains were obtained from the *Caenorhabditis* Genetics Center (CGC, University of Minnesota, Minneapolis, MN, USA). All nematodes were maintained at 20 °C on NGM agar plates seeded with ampicillin-resistant *E. coli* OP50 according to standard protocols.

### 4.2. Larval Development

The larval development assay was conducted on synchronized L1 worms until they reached the adult stage using an SMZ18 stereomicroscope equipped with a DS-Fi2 camera (Nikon Instruments Inc., Tokyo, Japan) as previously described [13]. Briefly, approximately 400 synchronized L1 worms were transferred onto a total of four NGM-OP50 plates per strain, resulting in approximately 100 worms per plate. Two of the plates were incubated at 20 °C, while the remaining two were incubated at 25 °C for a duration of four days. At 0, 5, 24, 29, 48, 53, 72, and 77 h after transferring the worms, photographs of 50 worms per strain (25 per plate) were taken using a Nikon SMZ18 stereomicroscope equipped with a DS-Fi2 camera (Nikon Instruments Inc., Tokyo, Japan). The length of each worm was measured using Nis Elements Documentation software version 4.00 (Nikon Instruments Inc., Tokyo, Japan). Subsequently, an estimation of the developmental stage of each worm at each time point (L1, L2, L3, early L4, late L4, young adult, or adult) was made based on its length and morphological characteristics.

### 4.3. Molting

Molting was studied in a conventional luminometer using the bioluminescence-based method previously published by our laboratory [33]. For this experiment, the strains COP1358 (*crt-1*(*knu378*)), JLV544 (*crt-1*(*jvp1*)), and KJ216 (*crt-1*(*jh101*)) were crossed with the reporter strain PE255 (*feIs5*[*sur-5p::luciferase::GFP + rol-6*(*su1006*)] X) until a homozygous strain for the *crt-1* mutations and the reporter construction was obtained.

### 4.4. Embryonic Cell Lineage Analysis

Embryonic cell lineage analysis was carried out by 4D microscopy (multi-focal time-lapse microscopy) as described in [34,35]. Briefly, gravid hermaphrodites were dissected, and 2- to 4-cell stage embryos were mounted on 4% agar pads in water and sealed with vaseline. Images on 30 focal planes (1 micron/section) were taken every 30 s for 12 h at 25 °C on a Leica DM6000 microscope fitted with DIC optics (Leica Microsystems GmbH, Wetzlar, Germany). The use of DIC optics allows cell tracing without using any dye or fluorescent markers that might alter cell cycle progression. The microscope was controlled with Time to Live software version 11_2010, and embryo lineages were analyzed with SIMI^®^ BioCell software version 4.0.149 (www.simi.com [36]).

### 4.5. Field Emission Scanning Electron Microscopy (FE-SEM)

To evaluate the effects of *crt-1* mutations on the cuticle morphology of *C. elegans* strains, worms at different larval stages were examined using Field Emission Scanning Electron Microscopy (FE-SEM).

Sample preparation followed the protocols described [37,38] with minor modifications. Briefly, worms from various larval stages grown at 20 °C on an NGM-OP50 plate were collected and washed with PBST. The worm pellets were then fixed by adding 20 µL of 2% glutaraldehyde and incubated overnight at room temperature (RT) with gentle agitation. After this period, the worms were washed again in PBST, and 1 mL of 1% osmium tetroxide dissolved in PBST was added to the tube in a chemical fume hood. After incubating for 30 min at RT, the tube was centrifuged, and the supernatant was removed using a micropipette. Finally, the samples were dehydrated in a series of ethanol solutions of increasing concentration to gradually displace the water from the sample. For this, 1 mL of a 35% ethanol solution diluted in PBST was added, and it was incubated at 4 °C for 30 min with gentle agitation. After this, the tube was centrifuged, and the liquid content was aspirated as much as possible. To the sediment, 1 mL of 50% ethanol was added. This process was repeated with a solution of 100% ethanol. Subsequently, the samples were dried by critical point drying with liquid carbon dioxide at 50 atm pressure and then mounted on aluminum SEM stubs and shadowed by cathodic sputtering with 16 nm gold. Samples were visualized using the Zeiss Sigma 300 VP scanning electron microscope (Carl Zeiss Industrielle Messtechnik GmbH, Oberkochen, Germany), obtaining images of the cuticle in the mouth, body, and tail regions.

### 4.6. Cuticle Resistance

The cuticle resistance was evaluated using the protocol described in [39] with minor modifications. Briefly, worms were grown at 20 °C on an NGM-OP50 plate. Subsequently, 5 µL of a sodium hypochlorite solution with 6–14% active chlorine (Sigma-Aldrich Co., St. Louis, MO, USA) were pipetted onto ten adults from each strain. Using a Nikon SMZ18 stereoscopic microscope equipped with a DS-Fi2 camera (Nikon Instruments Inc., Tokyo, Japan), the moment of the first cuticle rupture was recorded, and photos were taken after 1, 2, 3, 4, and 5 min of exposure to bleach.

### 4.7. Fertility

Gravid worms were synchronized with bleach and incubated for 48 h at 20 °C in M9. After this time, 200 L1 larvae were seeded on an NGM-OP50 plate and incubated at 20 °C until reaching the L4 stage. Once in this stage, 36 worms per strain were individually transferred to each of the wells of a 12-well plate with NGM (2 mL per well) and ampicillin-resistant *E. coli* OP50 (25 μL per well), and they were incubated at 25 °C until individuals from each strain stopped laying eggs. Every 24 h, each worm was transferred to a new well, and the number of eggs laid by the same worm on the previous day was counted. This assay allowed us to evaluate both the total number of eggs laid by each worm during its fertile life and the point at which the worms stopped laying eggs.

### 4.8. Resistance to Endoplasmic Reticulum (ER) Stress

The ER stress assay was carried out following the same procedure used in the larval development assay, but in this case, synchronized L1 worms were exposed to 25 °C on NGM-OP50 plates containing 2 μg/mL tunicamycin.

### 4.9. Fat Levels with Nile Red Staining

The levels of fat in *C. elegans* strains were quantified using Nile Red staining. To carry out this assay, gravid worms were synchronized and incubated at 20 °C and 25 °C on NGM-OP50 plates. Once the worms reached the L4 stage, they were collected and washed twice with PBST. Tubes were then incubated on ice for 15 min. After aspiration, worms were incubated at room temperature (RT) for 3 min in 200 μL of 40% isopropanol. Once the supernatant was removed, 150 μL of a freshly prepared 3 μg/mL Nile Red solution in 40% isopropanol was added. After 30 min at 20 °C with constant agitation, the supernatant was removed to stop the reaction with the dye. Subsequently, worms were washed with PBST and mounted on slides with 2% agarose. Images were then taken immediately using a Nikon Eclipse 80i fluorescence microscope (Nikon Instruments Inc., Tokyo, Japan) coupled with a CCD camera with a FITC filter (Ex 465–495; DM 505; BA 515–555) at 4× magnification (40× total magnification) under the same conditions and the same integration time. The emitted fluorescence was quantified using the ImageJ version 1.48v analysis program [40].

### 4.10. Statistics

Statistical analysis was performed using StataSE v12 (StataCorp LP, College Station, TX, USA) and GraphPad Prism v8.0.2 (GraphPad Software, San Diego, CA, USA). The significance level (α) was set at 0.05. Differences were considered as non-significant (ns) when *p* > 0.05, significant (*) when *p* < 0.05, very significant (**) when *p* < 0.01, and highly significant (***) when *p* < 0.001.

The normality of the residuals of all the quantitative variables analyzed was assessed first. If the normality conditions of the residuals were met, strains were compared using a one-way ANOVA test (if homogeneity of variances was assumed) or Welch’s test (if homogeneity of variances was not assumed), followed by multiple comparisons. Under these conditions, mean and standard deviation were used as descriptive statistics. If the normality conditions of the residuals were not met, the Kruskal–Wallis test (if distributions were similar) or the median test (if distributions were different) was conducted, followed by multiple comparisons. In this case, median and interquartile ranges were used as descriptive statistics.

For the qualitative variables, Pearson’s chi-square test or Fisher’s exact test was used to determine whether the strain affects the proportion of worms in each developmental stage, following Cochran’s rule. Finally, the day on which individuals of each strain stopped laying eggs was evaluated using the survival curves generated with the Kaplan–Meier method. The survival curves of the strains were compared with the log-rank test.

## 5. Conclusions

Overall, the findings collectively support the notion that mutations in *CALR* could have significant effects on MPNs as a consequence of a loss of function of the protein. The further exploration of these effects for the advancement of novel therapies in these patients holds considerable promise and interest.

## Data Availability

All relevant data can be found within the article and its Supplementary Materials. The datasets generated during the current study are available from the corresponding author upon request.

## Supplementary Materials

The following supporting information can be downloaded at: https://www.mdpi.com/article/10.3390/ijms252111606/s1.

## References

[1] Cerón J. (2023). *Caenorhabditis elegans* for research on cancer hallmarks. Dis. Models Mech..

[2] Sendoel A., Kohler I., Fellmann C., Lowe S.W., Hengartner M.O. (2010). HIF-1 antagonizes p53-mediated apoptosis through a secreted neuronal tyrosinase. Nature.

[3] Possik E., Jalali Z., Nouët Y., Yan M., Gingras M.C., Schmeisser K., Panaite L., Dupuy F., Kharitidi D., Chotard L. (2014). Folliculin regulates ampk-dependent autophagy and metabolic stress survival. PLoS Genet..

[4] Kyriakakis E., Markaki M., Tavernarakis N. (2015). *Caenorhabditis elegans* as a model for cancer research. Mol. Cell. Oncol..

[5] Kirienko N.V., Mani K., Fay D.S. (2010). Cancer models in *Caenorhabditis elegans*. Dev. Dyn..

[6] McGary K.L., Park T.J., Woods J.O., Cha H.J., Wallingford J.B., Marcotte E.M. (2010). Systematic discovery of nonobvious human disease models through orthologous phenotypes. Proc. Natl. Acad. Sci. USA.

[7] Tefferi A., Vannucchi A.M., Barbui T. (2024). Essential thrombocythemia: 2024 update on diagnosis, risk stratification, and management. Am. J. Hematol..

[8] Constantinescu S.N., Vainchenker W., Levy G., Papadopoulos N. (2021). Functional consequences of mutations in myeloproliferative neoplasms. Hemasphere.

[9] Pecquet C., Papadopoulos N., Balligand T., Chachoua I., Tisserand A., Vertenoeil G., Nédélec A., Vertommen D., Roy A., Marty C. (2023). Secreted mutant calreticulins as rogue cytokines in myeloproliferative neoplasms. Blood.

[10] Vadeikienė R., Jakštys B., Laukaitienė D., Šatkauskas S., Juozaitytė E., Ugenskienė R. (2024). The Role of Mutated Calreticulin in the Pathogenesis of *BCR-ABL1*-Negative Myeloproliferative Neoplasms. Int. J. Mol. Sci..

[11] Guijarro-Hernández A., Vizmanos J.L. (2021). A broad overview of signaling in *Ph*-negative classic myeloproliferative neoplasms. Cancers.

[12] Salati S., Genovese E., Carretta C., Zini R., Bartalucci N., Prudente Z., Pennucci V., Ruberti S., Rossi C., Rontauroli S. (2019). Calreticulin Ins5 and Del52 mutations impair unfolded protein and oxidative stress responses in K562 cells expressing CALR mutants. Sci. Rep..

[13] Guijarro-Hernández A., Eder-Azanza L., Hurtado C., Navarro-Herrera D., Ezcurra B., Novo F.J., Cabello J., Vizmanos J.L. (2023). Transcriptomic analysis reveals JAK2/MPL-independent effects of calreticulin mutations in a *C. elegans* model. Cells.

[14] Liu P., Zhao L., Loos F., Marty C., Xie W., Martins I., Lachkar S., Qu B., Waeckel-Énée E., Plo I. (2020). Immunosuppression by Mutated Calreticulin Released from Malignant Cells. Mol. Cell.

[15] Pronier E., Cifani P., Merlinsky T.R., Berman K.B., Somasundara A.V.H., Rampal R.K., LaCava J., Wei K.E., Pastore F., Maag J.L. (2018). Targeting the CALR interactome in myeloproliferative neoplasms. JCI Insight.

[16] Arshad N., Cresswell P. (2018). Tumor-associated calreticulin variants functionally compromise the peptide loading complex and impair its recruitment of MHC-I. J. Biol. Chem..

[17] Di Buduo C.A., Abbonante V., Marty C., Moccia F., Rumi E., Pietra D., Soprano P.M., Lim D., Cattaneo D., Iurlo A. (2020). Defective interaction of mutant calreticulin and SOCE in megakaryocytes from patients with myeloproliferative neoplasms. Blood.

[18] Eder-Azanza L., Navarro D., Aranaz P., Novo F.J., Cross N.C.P., Vizmanos J.L. (2014). Bioinformatic analyses of CALR mutations in myeloproliferative neoplasms support a role in signaling. Leukemia.

[19] Thiele J., Kvasnicka H.M., Orazi A., Gianelli U., Gangat N., Vannucchi A.M., Barbui T., Arber D.A., Tefferi A. (2023). The international consensus classification of myeloid neoplasms and acute leukemias: Myeloproliferative neoplasms. Am. J. Hematol..

[20] Harrison C.N., Mead A.J., Panchal A., Fox S., Yap C., Gbandi E., Houlton A., Alimam S., Ewing J., Wood M. (2017). Ruxolitinib *vs.* best available therapy for ET intolerant or resistant to hydroxycarbamide. Blood.

[21] Park B.J., Lee D.G., Yu J.R., Jung S.K., Choi K., Lee J., Lee J., Kim Y.S., Lee J.I., Kwon J.Y. (2001). Calreticulin, a calcium-binding molecular chaperone, is required for stress response and fertility in *Caenorhabditis elegans*. Mol. Biol. Cell.

[22] Wang W.A., Liu W.X., Durnaoglu S., Lee S.K., Lian J., Lehner R., Ahnn J., Agellon L.B., Michalak M. (2017). Loss of calreticulin uncovers a critical role for calcium in regulating cellular lipid homeostasis. Sci. Rep..

[23] Sawa M., Suetsugu S., Sugimoto A., Miki H., Yamamoto M., Takenawa T. (2003). Essential role of the *C. elegans* Arp2/3 complex in cell migration during ventral enclosure. J. Cell Sci..

[24] Szekely T., Wichmann B., Maros M.E., Csizmadia A., Bodor C., Timar B., Krenacs T. (2023). Myelofibrosis progression grading based on type I and type III collagen and fibrillin 1 expression boosted by whole slide image analysis. Histopathology.

[25] Delaval B., Lelièvre H., Birnbaum D. (2005). Myeloproliferative disorders: The centrosome connection. Leukemia.

[26] Prins D., Green A.R. (2020). Mutant CALR functions: Gains and losses. Blood.

[27] Van Duyn Graham L., Sweetwyne M.T., Pallero M.A., Murphy-Ullrich J.E. (2010). Intracellular calreticulin regulates multiple steps in fibrillar collagen expression, trafficking, and processing into the extracellular matrix. J. Biol. Chem..

[28] Ibarra J., Elbanna Y.A., Kurylowicz K., Ciboddo M., Greenbaum H.S., Arellano N.S., Rodriguez D., Evers M., Bock-Hughes A., Liu C. (2022). Type I but not type II calreticulin mutations activate the IRE1α/XBP1 pathway of the unfolded protein response to drive myeloproliferative neoplasms. Blood Cancer Discov..

[29] Yen K., Le T.T., Bansal A., Narasimhan S.D., Cheng J.X., Tissenbaum H.A. (2010). A comparative study of fat storage quantitation in nematode *Caenorhabditis elegans* using label and label-free methods. PLoS ONE.

[30] Pernes G., Flynn M.C., Lancaster G.I., Murphy A.J. (2019). Fat for fuel: Lipid metabolism in haematopoiesis. Clin. Transl. Immunol..

[31] Mesaeli N., Nakamura K., Zvaritch E., Dickie P., Dziak E., Krause K.H., Opas M., MacLennan D.H., Michalak M. (1999). Calreticulin is essential for cardiac development. J. Cell Biol..

[32] Guo L., Nakamura K., Lynch J., Opas M., Olson E.N., Agellon L.B., Michalak M. (2002). Cardiac-specific expression of calcineurin reverses embryonic lethality in calreticulin-deficient mouse. J. Biol. Chem..

[33] Guijarro-Hernández A., Hurtado C., Martínez-Irujo J.J., Vizmanos J.L. (2023). Monitoring *Caenorhabditis elegans* molting in a conventional luminometer. MethodsX.

[34] Mora-Lorca J.A., Sáenz-Narciso B., Gaffney C.J., Naranjo-Galindo F.J., Pedrajas J.R., Guerrero-Gómez D., Dobrzynska A., Askjaer P., Szewczyk N.J., Cabello J. (2016). Glutathione reductase *gsr-1* is an essential gene required for *Caenorhabditis elegans* early embryonic development. Free Radic. Biol. Med..

[35] Escrich V., Ezcurra B., Gómez-Orte E., Romero-Aranda C., Miranda-Vizuete A., Cabello J. (2020). 4D Microscopy: Unraveling *Caenorhabditis elegans* embryonic development using Nomarski microscopy. J. Vis. Exp..

[36] Schnabel R., Hutter H., Moerman D., Schnabel H. (1997). Assessing normal embryogenesis in *Caenorhabditis elegans* using a 4D microscope: Variability of development and regional specification. Dev. Biol..

[37] Woodruff G.C., Knauss C.M., Maugel T.K., Haag E.S. (2014). Mating damages the cuticle of *C. elegans* hermaphrodites. PLoS ONE.

[38] Blaxter M.L. (1993). Cuticle surface proteins of wild type and mutant *Caenorhabditis elegans*. J. Biol. Chem..

[39] Calvo A.C., Pey A.L., Ying M., Loer C.M., Martinez A. (2008). Anabolic function of phenylalanine hydroxylase in *Caenorhabditis elegans*. FASEB J..

[40] Schneider C.A., Rasband W.S., Eliceiri K.W. (2012). NIH Image to ImageJ: 25 years of image analysis. Nat. Methods.

