# Supplementary material for: Myeloproliferative Neoplasm-like Mutations of Calreticulin Induce Phenotypes Associated with Calreticulin Dysfunction in C. elegans
Source: Int J Mol Sci. 2024 Oct 29;25(21):11606. doi: 10.3390/ijms252111606 (PMC11546369; doi:10.3390/ijms252111606)
Supplement: Supplementary file 1 [file ijms-25-11606-s001.zip › ijms-3262536-supplementary.pdf]

Supplementary Material

Myeloproliferative Neoplasm-like Mutations of Calreticulin Induce Phenotypes Associated with Calreticulin Dysfunction in *C. elegans*

Ana Guijarro-Hernández [1], Cristina Hurtado [1], Estibaliz Urizar-Compains [1], Begoña Ezcurra [2], Alberto Galiana-Sáenz [1,2], Enrique Baquero [3,4], Juan Cabello [2], José Luis Vizmanos [1,*]

[1] Department of Biochemistry and Genetics, School of Sciences, University of Navarra, 31008 Pamplona, Spain
[2] Center for Biomedical Research of La Rioja (CIBIR), 26006 Logroño, Spain
[3] Department of Environmental Biology, School of Sciences, University of Navarra, 31008 Pamplona, Spain
[4] Institute for Biodiversity and Environment BIOMA, University of Navarra, 31008 Pamplona, Spain
* Correspondence: jlvizmanos@unav.es

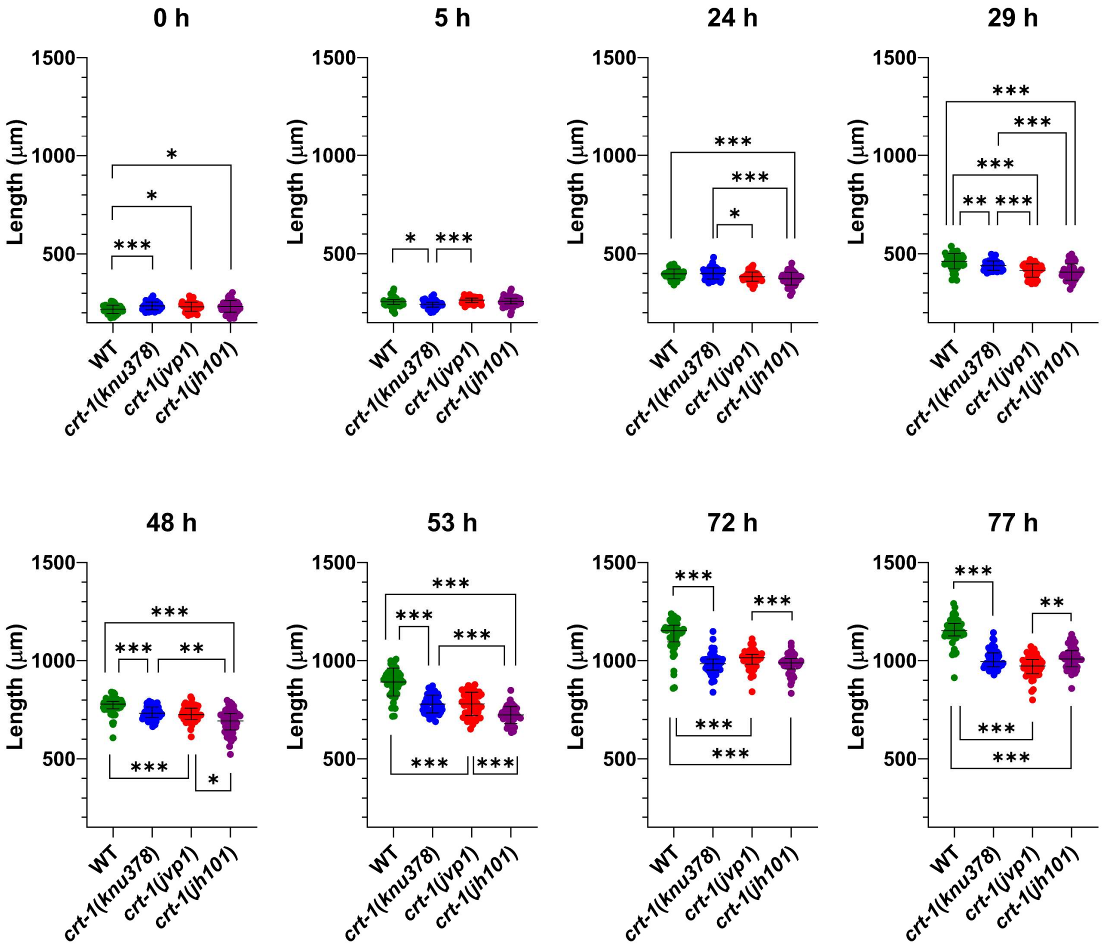

Figure S1. Comparison of the length of *crt-1* mutants and wild-type worms at 0, 24, 29, 48, 53, 72, and 77 h of growth at 20 °C. In all cases, the mean (0, 24, 29, and 53 h) or the median (48, 72, and 77 h) of the lengths obtained for 50 worms per strain is represented along with their standard deviation or interquartile range, respectively. The choice between representing a statistic or another varied depending on the normality of the residuals and consequently, on the statistical test used (parametric or non-parametric) for data analysis. Differences were considered significant (*) when $p < 0.05$, very significant (**) when $p < 0.01$, and highly significant (***) when $p < 0.001$.

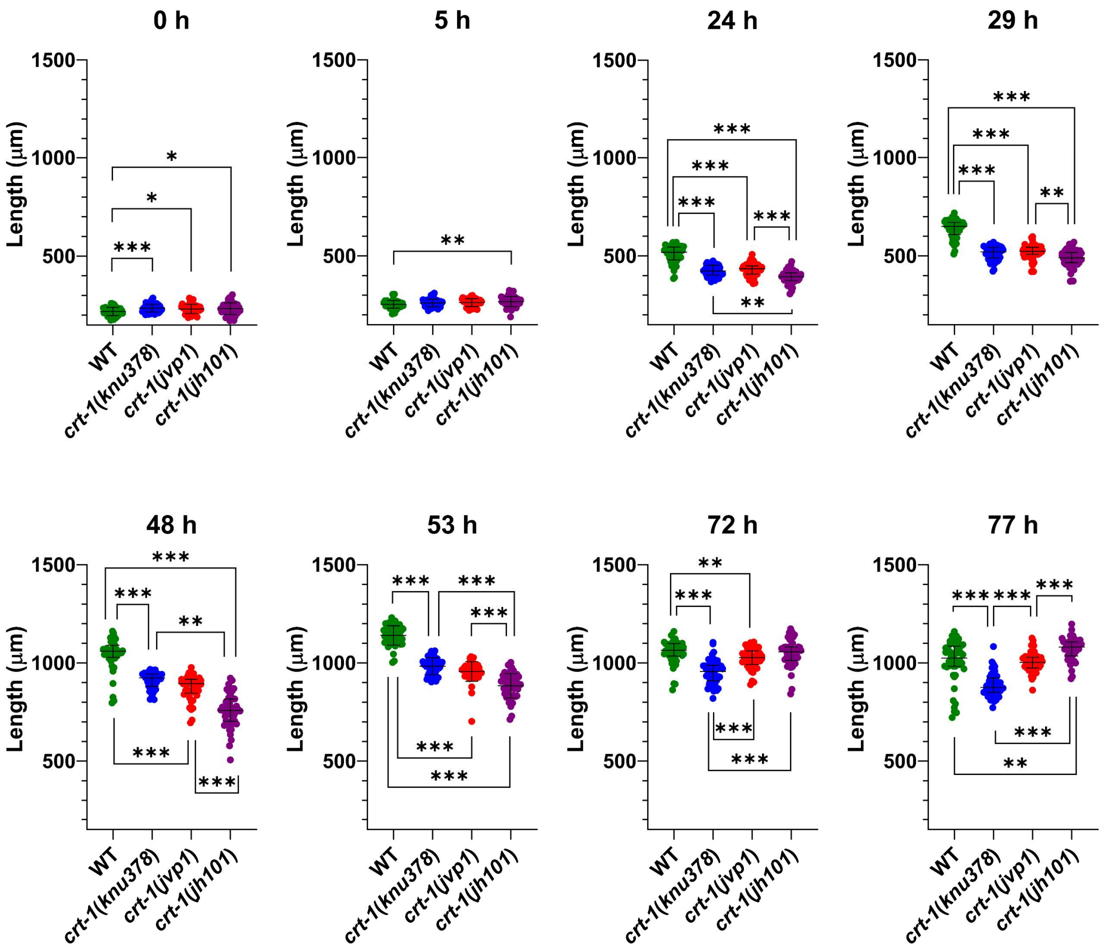

Figure S2. Comparison of the length of *crt-1* mutants and wild-type worms at 0, 24, 29, 48, 53, 72, and 77 h of growth at 25 °C. The mean (0, 5, and 53 h) or the median (24, 29, 48, 72, and 77 h) of the lengths obtained for 50 worms per strain is represented along with their standard deviation or interquartile range, respectively. The choice between representing one statistic or the other varied depending on the normality of the residuals and consequently, on the statistical test used (parametric or non-parametric) for data analysis. Differences were considered significant (*) when $p < 0.05$, very significant (**) when $p < 0.01$, and highly significant (***) when $p < 0.001$.

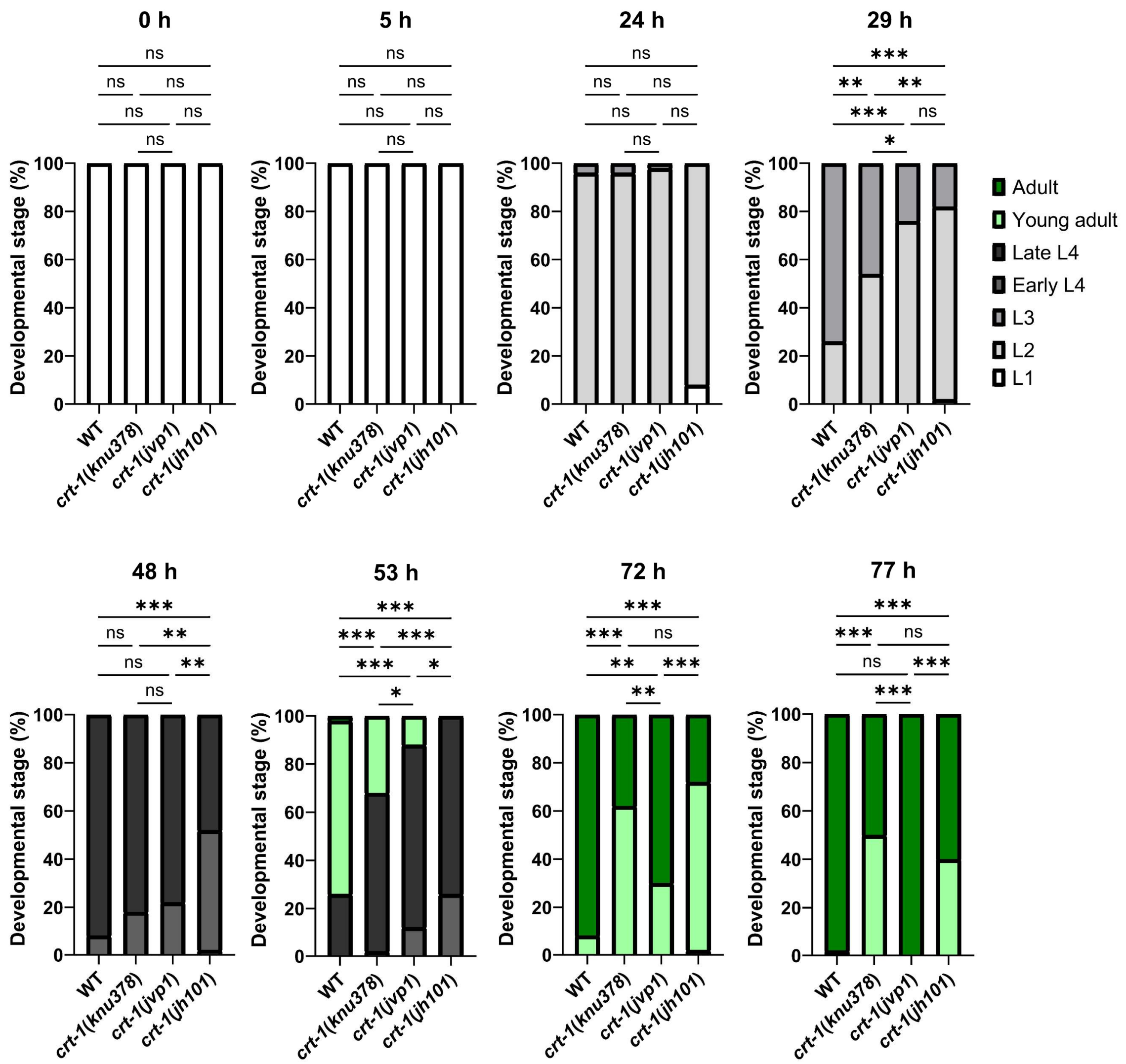

Figure S3. Comparison of larval stages in which *crt-1* mutants and wild-type worms are at 0, 24, 29, 48, 53, 72, and 77 h of growth at 20 °C. The percentage of worms in each larval stage (L1, L2, L3, early L4, late L4, young adult, or adult) is represented out of a total of 50 worms analyzed per strain at each time point evaluated. Differences were considered significant (*) when $p < 0.05$, very significant (**) when $p < 0.01$, and highly significant (***) when $p < 0.001$.

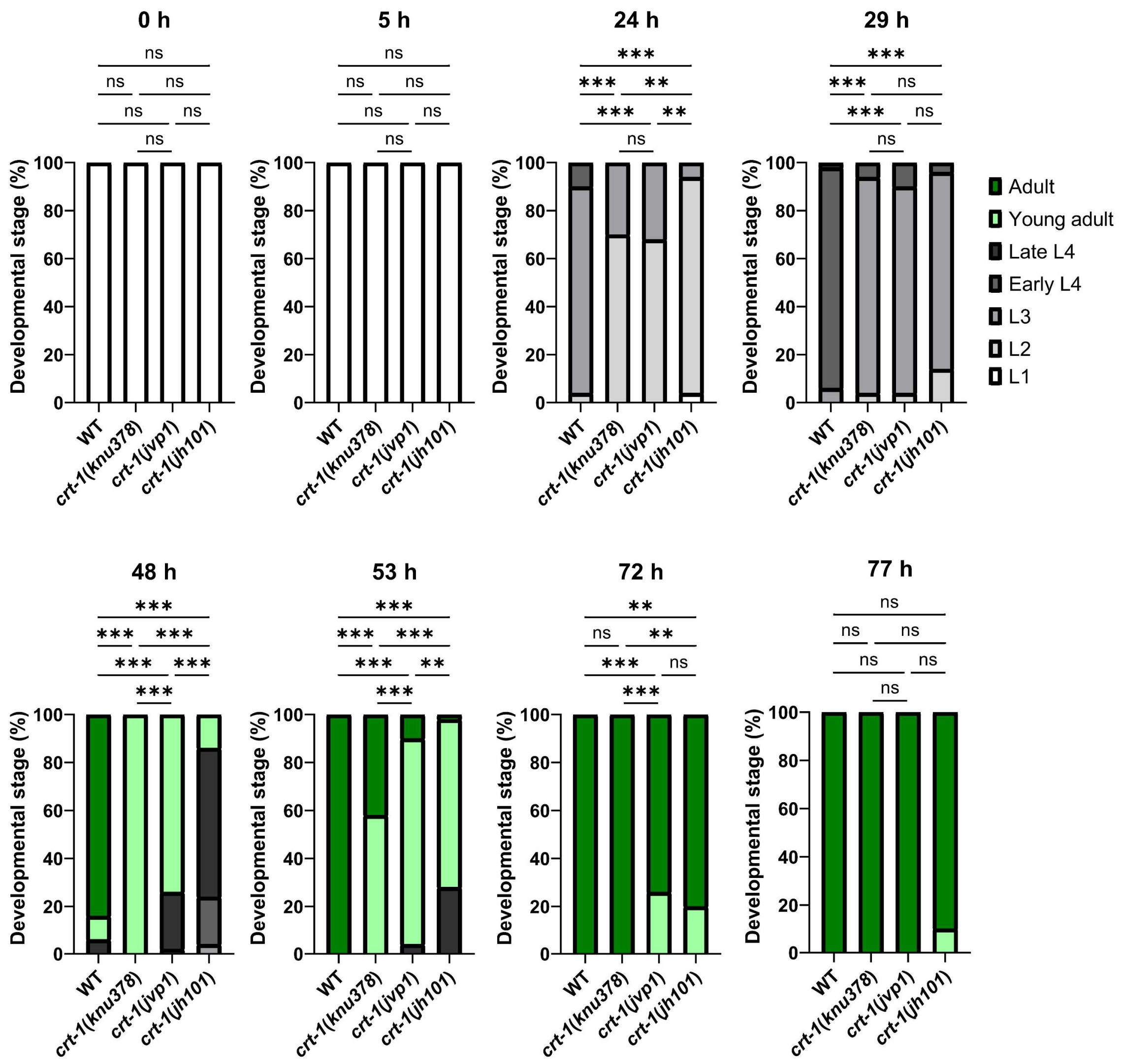

Figure S4. Comparison of larval stages in which *crt-1* mutants and wild-type worms are at 0, 24, 29, 48, 53, 72, and 77 h of growth at 25 °C. The percentage of worms in each larval stage (L1, L2, L3, early L4, late L4, young adult, or adult) is represented out of a total of 50 worms analyzed per strain at each time point evaluated. Differences were considered significant (*) when $p < 0.05$, very significant (**) when $p < 0.01$, and highly significant (***) when $p < 0.001$.

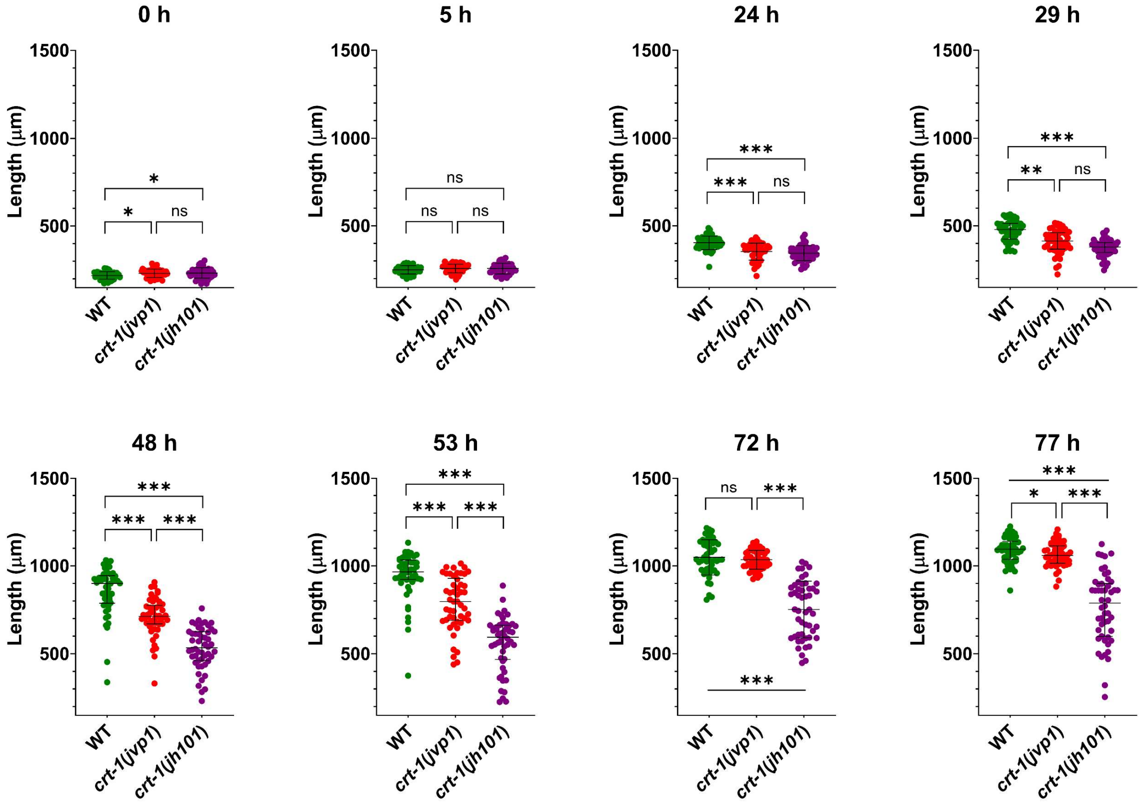

Figure S5. Comparison of the length of *crt-1* mutants and wild-type worms at 0, 24, 29, 48, 53, 72, and 77 h of growth at 25 °C in the presence of 2 µg/µL of tunicamycin. The mean (0, 5, 24, and 72 h) or the median (29, 48, 53, and 77 h) of the lengths obtained for 50 worms per strain is represented along with their standard deviation or interquartile range, respectively. The choice between representing one statistic or the other varied depending on the normality of the residuals and consequently, on the statistical test used (parametric or non-parametric) for data analysis. Differences were considered significant (*) when $p < 0.05$, very significant (**) when $p < 0.01$, and highly significant (***) when $p < 0.001$.

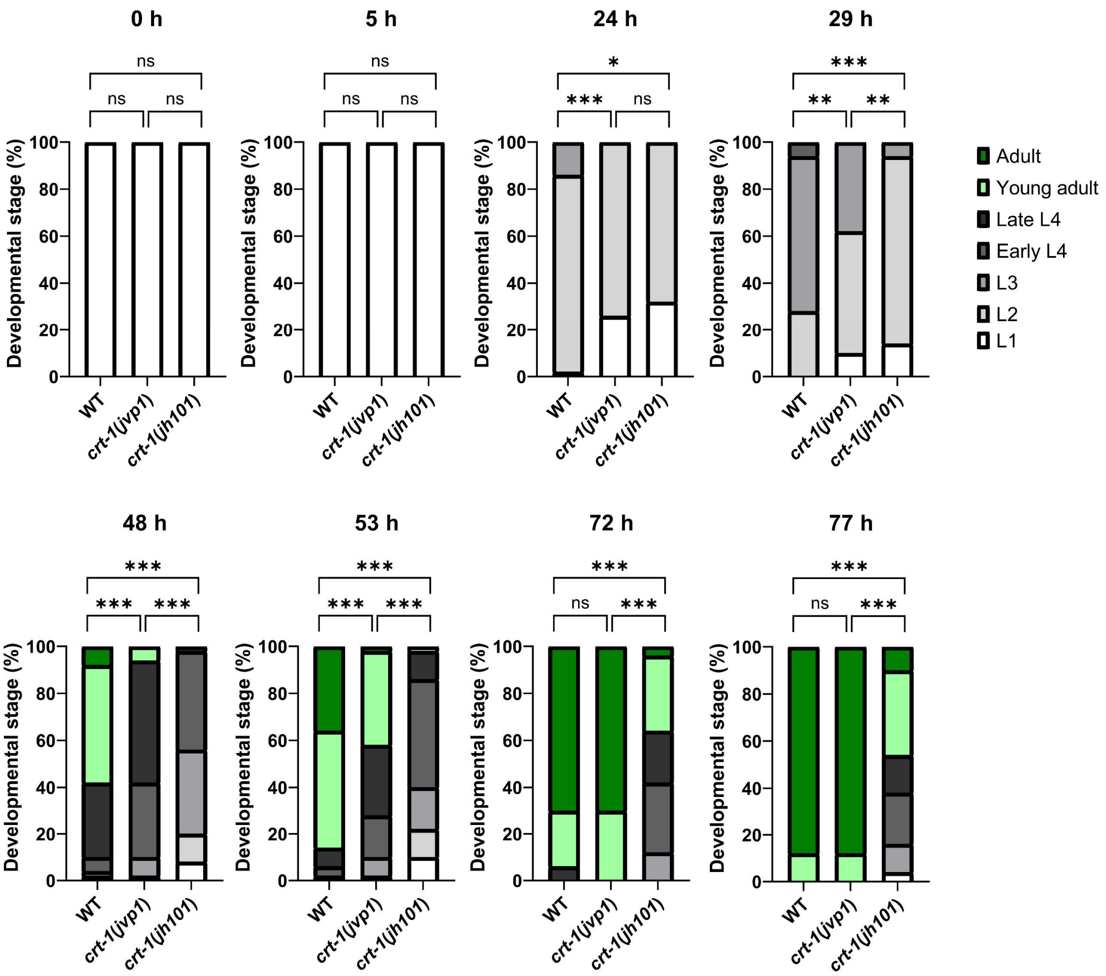

Figure S6. Comparison of larval stages in which *crt-1* mutants and wild-type worms are at 0, 24, 29, 48, 53, 72, and 77 h of growth at 25 °C in the presence of 2 µg/µL of tunicamycin. The percentage of worms in each larval stage (L1, L2, L3, early L4, late L4, young adult, or adult) is represented out of a total of 50 worms analyzed per strain at each time point evaluated. Differences were considered significant (*) when $p < 0.05$, very significant (**) when $p < 0.01$, and highly significant (***) when $p < 0.001$.
